# Growth Factor Independence-1 (GFI-1) Gene Expression in Hematopoietic Stem Cell Lineage Differentiation in Low Birth Weight Newborns Compared With Normal Birth Weight Newborns at Term Pregnancy

**DOI:** 10.7759/cureus.50696

**Published:** 2023-12-17

**Authors:** Najma Eram, Shikha Sachan, Jigyasa Singh, Shreya, Utkarsh Dwivedi, Doli Das, Geeta Rai, Mamta Rajan

**Affiliations:** 1 Obstetrics and Gynaecology, Institute of Medical Sciences, Banaras Hindu University (BHU), Varanasi, IND; 2 Molecular and Human Genetics, Institute of Medical Sciences, Banaras Hindu University (BHU), Varanasi, IND

**Keywords:** normal birth weight, growth factor independence 1, low birth weight, cdna, pcr

## Abstract

Introduction

Low birth weight (LBW), which is a risk factor for noncommunicable diseases throughout life, is a significant public health concern. In addition to regulating myeloid cell differentiation and proliferation, a transcriptional repressor identified as growth factor independence-1 (GFI-1) is essential for hematopoietic stem cell maintenance and self-renewal. The current study was designed to compare the expression of the GFI-1 gene in the differentiation of hematopoietic stem cells in newborns with LBW and those with normal birth weight (NBW).

Methods

A prospective comparative analytical study was carried out from September 2019 to September 2021 after obtaining Institute Ethical Committee approval at a tertiary care center in north India.

The GFI-1 gene expression levels in 50 cord blood samples from women with term gestation and LBW newborns (<2500 grams) were measured using quantitative real-time polymerase chain reaction (RT-PCR) and compared to gene expression levels in 50 cord blood samples from women with term gestation and NBW newborns (≥2500 grams). The data were analyzed using IBM SPSS statistics software version 24.0 (IBM Corp., Armonk, NY).

Results

The median GFI-1 expression in LBW newborns is 3.1, whereas among NBW newborns it is 9.39. The difference is significant (P <0.001). The level of GFI-1 gene expression in LBW newborns was correlated with their birth weight. The coefficient of correlation was found to be weakly positive (r = 0.223). The birth weight of NBW newborns was correlated to the level of expression of the GFI-1 gene, which was found to be positively correlated (r = 0.332).

Conclusion

The levels of the GFI-1 gene and newborn birth weight were compared in LBW infants, which were weakly positively correlated. The level of GFI-1 gene expression at birth was compared to the birth weight of NBW newborns, which was positively correlated.

## Introduction

The transcription-restrictor factor, growth factor independence-1 (GF-1) is required for multilineage hematopoietic stem cell (HSC) differentiation as well as neuronal development [1]. Cytokines that regulate both the acquired and innate immune systems quickly stimulate GFI-1 expression. The GFI-1 gene plays a significant role in a wide variety of disorders and is a lineage-specific transcription factor that, in turn, regulates the expression of genes involved in maintaining homeostasis [1].

During an in vitro screen for variables that promote the transition of a leukemia T-cell line to interleukin (IL)-2-independent growth, GFI-1 was originally discovered as the target gene of an insertion locus of the Moloney murine leukemia virus [1,2]. Following its discovery, a paralogue, GFI-1B, was also identified. After extensive research, it is now known that both GFI-1 and GFI-1B are transcription factors that have a crucial role in hematopoiesis. The DNA sequence TAAATCAC(A/T)GCA is bound by the DNA-binding protein GFI-1, which possesses six C2H2-type, C-terminal zinc finger motifs. Position- and orientation-independent active transcriptional repression activity by GFI-1 depends on a 20-amino acid N-terminal repressor domain that is coincident with a nuclear localization motif. The amino acid sequence of this domain is nearly identical in GFI-1B and similar in the insulinoma-associated protein IA-1, the homeobox protein GSH-1, the ovo-like proteins, and all the vertebrate members of the snail/slug family of mesoderm-determining transcription factors (snail + GFI-1 = SNAG domain). Deletion or mutation of the first seven amino acids in the GFI-1 or GFI-1B SNAG domain destroys the repression activity of either protein. Notably, mutation of the proline at position 2 to alanine (P2A) in GFI-1 significantly impaired repression function without disturbing nuclear localization [2].

Mutations of GFI-1 in humans were recently shown to correlate with severe congenital neutropenia (SCN) and non-immune chronic idiopathic neutropenia in adults (NI-CINA). Interestingly, GFI-1 is also expressed and is oncogenic in human small-cell lung cancer (SCLC), the deadliest neuroendocrine tumor [3].

Quiescent HSCs have the extraordinary ability to self-renew indefinitely. Many hematological malignancies originate in stem cells; therefore, understanding the processes that regulate their self-renewal, quiescence, and differentiation is important. Previous research has established that GFI-1 helps maintain HSC quiescence [4, 5]. Recently, the role of the tumor suppressor p53 in preserving HSC quiescence has been elucidated [6]. Hematopoietic stem cell numbers are higher in p53-deficient mice, but the proportion of cells with a quiescent state is lower. It is noteworthy that GFI-1 was found to be a p53 target gene in HSCs. The reduced HSC quiescence seen in p53-null mice may be partially explained by the down-regulation of GFI-1 expression in p53-null HSCs. This finding suggests that p53 directly regulates GFI-1 to control HSC quiescence and that GFI-1 is part of a network of intrinsic factors that control HSC destiny [5, 6].

The most apparent symptoms of GFI-1 deficiency appear during neutrophil maturation. It has been established that SCN and NI-CIN are caused in people by uncommon hereditary mutations in the GFI-1 gene [7, 8]. Decreased GFI-1 expression also leads to less secondary granule protein expression, which may play a role in the onset of a neutrophil-specific granule deficit. Mice lacking GFI-1 or expressing the mutant form of GFI-1 observed in SCN patients develop symptoms comparable to those seen in humans with SCN [9]. This is strong evidence that the mutated version of GFI-1 that causes SCN works in a dominant-negative way over the normal version.

Lineage destiny decisions between granulocyte and monocyte/macrophage development are mediated in the myeloid compartment by a regulatory network in which GFI-1 plays a role. In this subcellular location, GFI-1 inhibits PU.1's transcriptional activity. In contrast to GFI-1, which encourages granulocytic differentiation, PU.1 promotes monocytic differentiation. By competing for DNA binding in the promoters of target genes, GFI-1 and PU.1 are able to suppress each other's activity [10, 11]. On the other hand, GFI-1 inhibits PU.1 by directly repressing the PU.1 gene and by interfering with PU.1 function via a protein-protein interaction [12]. Contrarily, PU.1 promotes the production of transcription factors that suppress the expression of the GFI-1 gene. As a result, the neutropenia in SCN patients may be explained by the loss of GFI-1 function tipping the scales in favor of PU.1-mediated monocytic development. Moreover, this implies that patients with GFI-1 mutations may experience the activation of genes that GFI-1 normally turns off [11, 12].

The GFI-1 paralogue, GFI-1B, is extensively expressed in the progenitors of erythroid and megakaryocytic cells. The expression of GFI-1 in these cells is virtually nonexistent. Animals deficient in GFI-1B do not survive embryonic development due to a failure in definitive erythropoiesis, restricting further investigation into the potential roles of GFI-1B in further hematopoietic stages. Overexpression and silencing of GFI-1B in distinct immature precursor subsets have been used to avoid embryonic lethality. Silencing GFI-1B in CD34+ cells led to a reduction in progenitor cell proliferation and an inhibition of differentiation into erythroblasts and megakaryocytes, as seen below [13]. In contrast, overexpression of GFI-1B in human CD34+ cells increased the number of pro-erythroblasts but did not lead to erythroid commitment or prevent further differentiation. Nevertheless, in CD 36+/glycophorin A (GPA)-erythroid precursors, forced GFI-1B expression suppressed proliferation and prompted differentiation [14]. This suggests that GFI-1B, at different points in erythropoiesis, induces contrasting phenotypes. Loss of GFI-1B expression does not completely silence GFI-1 expression in immature CD34+ cells. Erythropoiesis and megakaryopoiesis are not recovered by GFI-1 expression [13,14]. This demonstrates that GFI-1 cannot compensate for the loss of GFI-1b during erythroid development.

In addition to the myeloid lineage, GFI-1 also plays a role in the development of the lymphoid lineage. From early common lymphoid progenitors to mature functional T cells, GFI-1 is required at multiple points in the T-cell life cycle. Absolute numbers of T lymphocytes are reduced in patients with SCN caused by mutations in the GFI-1 gene. Loss of GFI-1 function has a moderate impact on the growth of T helper 1 (Th1) cells but has a devastating impact on Th2 cells [15,16].

Mature B cells originate from common lymphoid progenitors in the bone marrow, where they progress through several stages of differentiation. When stimulated, B cells release antibodies targeting a particular target. The expression of GFI-1 is highest in immature B cells and declines with B cell maturation [17]. Mice deficient in GFI-1 had fewer B-cell precursors than wild-type animals [18]. Ectopic expression of GFI-1 can repair the significantly defective B-cell differentiation and proliferation of GFI-1-null cells in vitro. Yet, the number of developing B cells is boosted when GFI-1 is artificially expressed in multipotent progenitor cells. Expression of GFI-1 promotes B-cell development over myeloid development by inhibiting the transcription factor PU.1, but in myeloid development, it promotes neutrophil over macrophage development [19].

A low birth weight (LBW) is defined as a birth weight of less than 2500 grams, regardless of gestational age [20]. More than 20 million infants worldwide, representing 15.5% of all births, are born with LBW, 95.6% of them in developing countries [21]. Low birth weight has been associated with higher probabilities of infection, malnutrition, and handicapped conditions during childhood, as well as mental deficiencies and problems related to behavior and learning. Children who survive LBW have a higher incidence of diseases, retarded cognitive development, and poor nutrition. Low birth weight infants are classified as having a very low birth weight (VLBW: <1.5-1.0 kg) or an extremely low birth weight (ELBW: <1 kg). The primary causes of LBW are premature birth (being born before 37 weeks of gestation) and the mothers of LBW babies belonging to <19 and ≥30-year age groups [22]. Also, the risk of LBW newborns in low-income countries is due to intrauterine growth retardation (IUGR). Maternal health factors like proper nutrition and weight gain are also linked with fetal weight gain and birth weight. The mother should avoid alcohol, cigarettes, and illicit drugs, which can contribute to poor fetal growth [23].

With this background, the present study was planned to study the expression of the GFI-1 gene in HSC differentiation in LBW newborns vs. NBW newborns.

## Materials and methods

The present study was carried out in the Department of Obstetrics and Gynecology in association with the Department of Molecular and Human Genetics at the Banaras Hindu University, Varanasi, Uttar Pradesh. Ethical approval was obtained from the Institute Ethical Committee, Institute of Medical Sciences, Banaras Hindu University, Uttar Pradesh, India (reference number: DEAN/2020EC/2053).

The data were analyzed using IBM SPSS version 24 (IBM Corp., Armonk, NY). The assessment of significance was done using the Chi-squared test ( χ2 test) and Fisher's exact test. A P-value<0.05 was considered statistically significant. A written informed consent was obtained from all the participants before the start of the study.

Study design

This was a prospective comparative analytical study.

Aim and objective of the study

Study of the expression of the GFI-1 gene in hematopoietic stem cell differentiation in low birth weight newborns compared with normal birth weight newborns.

Inclusion criteria

All the singleton pregnancies after 37 completed weeks of gestational age, whether booked or unbooked, were included for the study purpose.

Cases

Women with term gestation and low birth weight newborns (<2500 grams) who had a normal vaginal delivery or cesarean delivery, with a sample size of 50 were included.

Control

Women with term gestation and normal birth weight newborns (≥ 2500 grams) who had a normal vaginal delivery or cesarean delivery, with a sample size of 50 were included.

Exclusion criteria

Multiple gestations, diabetes mellitus, infectious diseases recognized in pregnancy, preeclampsia, preterm labor, Mullerian anomaly, IUGR, and fetal anomalies.

Gestational age was stabilized based on the last menstrual period and/or ultrasound evaluation prior to 20 weeks.

A total of 50 women with singleton pregnancies after 37 completed weeks of gestation with low birth weight newborns and 50 women with normal birth weight newborns were recruited for the study. Written and informed consent was obtained from all the participants prior to enrollment.

For each patient, a detailed sociodemographic and medical history were taken. Clinical examinations and routine relevant investigations were done. Each baby was examined for sex, birth weight, APGAR score at one minute, and NICU admission.

Collection of blood samples

From the fetuses of the cases and controls, 5 ml of cord blood was extracted and placed in sterile vials.

Isolation of ribonucleic acid (RNA) from the blood sample

Microcentrifuge tubes, pipette tips, and polymerase chain reaction (PCR) tubes were treated with 0.05% diethylpyrocarbonate (DEPC) before the isolation of RNA. One ml of cord blood sample was taken in a microcentrifuge tube, and 1 ml of trizol was added and vortexed vigorously. After that, keeping it for five minutes at room temperature, 200 μl of chloroform was added and mixed by inverting for 15 seconds and was kept for 15 minutes at room temperature. Centrifugation was done at 12,000 g for 15 minutes at 4 °C. The upper aqueous phase was transferred to a fresh microcentrifuge tube; 500 μl of isopropanol was added, mixed gently, and kept for 10 minutes at room temperature. Centrifugation was done at 12,000 g for 10 minutes at 4 °C. The RNA was precipitated as a pellet, and the supernatant was discarded. The pellet was washed with 1 ml of 75% ethanol (ice cold) and kept for five seconds, followed by repeat centrifugation at 12000 g for 5 minutes at 4 ˚C. The ethanol was removed, and the pellet was air-dried. To dissolve the pellet, 10 μl of nuclease-free water was added.

Preparation of copy DNA (cDNA) from the RNA sample

The sample of the RNA, deoxyribonuclease (DNase) buffer, DNase enzyme (1U), 25 mM ethylenediaminetetraacetic acid, 10X reverse transcription (RT) buffer, 25X deoxynucleotide triphosphate (dNTPs), 10X RT random primers, RT enzyme, ribonuclease (RNase) inhibitor (10U/μl), Milli-Q (MQ) Water, and ice were used.

To prepare cDNA from an RNA sample using the Applied Biosystems (ABI) protocol, we set up reactions one and two as follows:

Reaction One

To prepare the template for the reaction, the following components were mixed:

RNA (2.5 micrograms): added according to the concentration; DNase Buffer (10X): 1 microliter; DNase I (1 U): 0.5 microliter; and RNase inhibitor (10 U/microliter): 1 microliter.

Using MQ water, the volume was finally made up to 10 microliters. The reaction was incubated at 37 ˚C for 15 minutes, followed by 1 microliter of 25 mM EDTA (DEPC-treated pH 8.0) being added and mixed properly, and the reaction was incubated at 65 ˚C for 15 minutes, followed by an ice chill for a few minutes. To this reaction mixture, a 2X RT master mix of the cDNA preparation kit was added.

Reaction Two

The master mixture was prepared using 2 microliters of 10x RT buffer, 0.8 microliters of 25x dNTP, 2 microliters of 10x RT random primer, 1 microliter of RT enzyme, 1 microliter of RNase inhibitor, and 3.2 microliters of nuclease-free water.

Reaction one and reaction two were added, mixed properly, and incubated in four steps. The temperatures were 25 ˚C, 37 ˚C, 85 ˚C, and 4 ˚C, and the times of incubation were 10, 120, five, and one minute, respectively, in the four different stages.

Real-time PCR

The cDNA samples, SYBR Green Master Mix, GFI-1 and β-actin primers (β-actin is used for normalization), and nuclease-free water were used. Reactions were set up in duplicates for each gene with all samples, and then 1.5 microliters of cDNA sample were added to each tube containing the reaction master mixture of forward primer (0.3 microliters), reverse primer (0.3 microliters), PCR master mixture (7.5 microliters), and nuclease-free water (5.4 microliters).

The PCR reaction steps

Initial activation of the PCR reaction was for five minutes at 95 ˚C, denaturation for 30 seconds at 95 ˚C, combined annealing at 60 ˚C, and extensions at 72 ˚C for 30 seconds for a total of 40 cycles.

## Results

All the sociodemographic variables are depicted in Table 1.

**Table 1:** Demographic variables and newborn properties LBW: low birth weight; STD: sexually transmitted diseases; SVD: spontaneous vaginal delivery; LSCS: lower (uterine) segment cesarean section; NICU: neonatal intensive care unit

Variable		Case (n=55), n (%)	Control (n=52), n (%)	P-value
Age (years)	18-20	6 (12%)	0	0.092
	20-25	14 (28%)	16 (32%)	
	26-30	15 (30%)	18 (36%)	
	>30	15 (20%)	16 (32%)	
Parity	Nulliparous	14 (28%)	17 (34%	<0.001
	Multiparous	36 (72%)	33 (66%)	
Educational level	Illiterate	15 (30%)	13 (26%)	0.862
	High school	18 (36%)	19 (38%)	
	Intermediate	7 (14%)	6 (12%)	
	Graduate	0	1 (2%)	
	Postgraduate	10 (20%)	11 (22%)	
Socioeconomic status	Low	32 (64%)	4 (8%)	<0.001
	Middle	14 (28%)	25 (50%)	
	Upper	4 (8%)	21 (42%)	
History of previous LBW baby		15 (30%)	5 (10%)	0.012
History of STD in the mother		5 (10%)	2 (4%)	0.240
History of systemic infection in the mother		7 (14%)	3 (6%)	0.182
History of vaginal bleeding in the first trimester		4 (8%)	3 (6%)	0.696
Smoking		1 (2%)	0	0.315
Alcohol/ caffeine intake		2 (4%)	1 (2%)	0.558
Stress		9 (18%)	3 (6%)	0.065
Weight gain during pregnancy	Upto 6 kgs	28 (56%)	0	0
	7-8 kgs	18 (36%)	1 (2%)	
	8-10 kgs	4 (8%)	49 (98%)	
Mode of delivery	SVD	21 (42%)	24 (48%)	1.00
	LSCS	29 (58%)	26 (52%)	
APGAR at 1 minute	5	3 (6%)	0	0.021
	6	1 (2%)	2 (4%)	
	7	8 (16%)	1 (2%)	
	8	38 (76%)	47 (94%)	
NICU admission		16 (32%)	9 (18%)	0.175
Newborn survival		49 (98%)	50 (100%)	0

In our study, the majority of patients in the case group (30%) and control group (36%), respectively, were aged 26-30 years. Multiparous women were predominant in both the case (72%) and control (66%) groups. Thirty-six percent of patients with LBW had high school as their educational status and 30% were illiterate, while in the control group, 38% had high school as their educational status and 26% were illiterate. In patients with LBW, the majority belonged to the lower class, whereas in the control group, 50% belonged to the middle class and 42% to the upper class, according to the Kuppuswamy scale. Thirty percent of females with LBW had a previous history of LBW, while 10% of females with a normal birth weight had a previous history of LBW. Ten percent of the females in the case group, whereas only 4% of the females in the control group, had a history of sexually transmitted disease (STD) infection. The history of systemic infection was present in 14% of females in the case group, while it was only 6% in the control group, but that was not significant.

A history of vaginal bleeding was present in 8% of females in the case group and 6% of the control group. The habit of smoking was present in only 2% of females in the case group, whereas none had it in the control group. Four percent of the participants in the case group had excess caffeine intake, whereas, in the control group, 2% had excess caffeine intake, but that was not significant. Stress factors were present in 18% of the case group, whereas in the control group, only 6% had stress factors. The majority of the patients in the case group (56%) had weight gains up to 6 kg, and 98% of patients in the control group had weight gains of 9 kg or more. Sixty percent of patients in the case group had a BMI between 18.5 and 24.99 kg/m2, whereas the majority of control group patients (56%) had a BMI of 25 or above.

In our study, 42% of the females in the case group delivered vaginally and 58% delivered through a cesarean section, while in the control group, 48% delivered vaginally, and 52% delivered through a cesarean section. Female birth predominance was in the case group (56%), and male birth predominance was in the control group (52%).

In our study, 76% of neonates in the case group had a one-minute APGAR score of eight, whereas in the control group, a one-minute APGAR score of eight was seen in 94% of neonates. Six percent of LBW babies had an APGAR score of five, 2% had an APGAR score of six, and 16% had an APGAR score of seven at one minute. Thirty-two percent of babies with LBW required admission to the neonatal intensive care unit (NICU), and 18% of babies with NBW required NICU admission. Despite the poor scores, 98% of LBW babies survived, while only 2% died.

**Figure 1:**
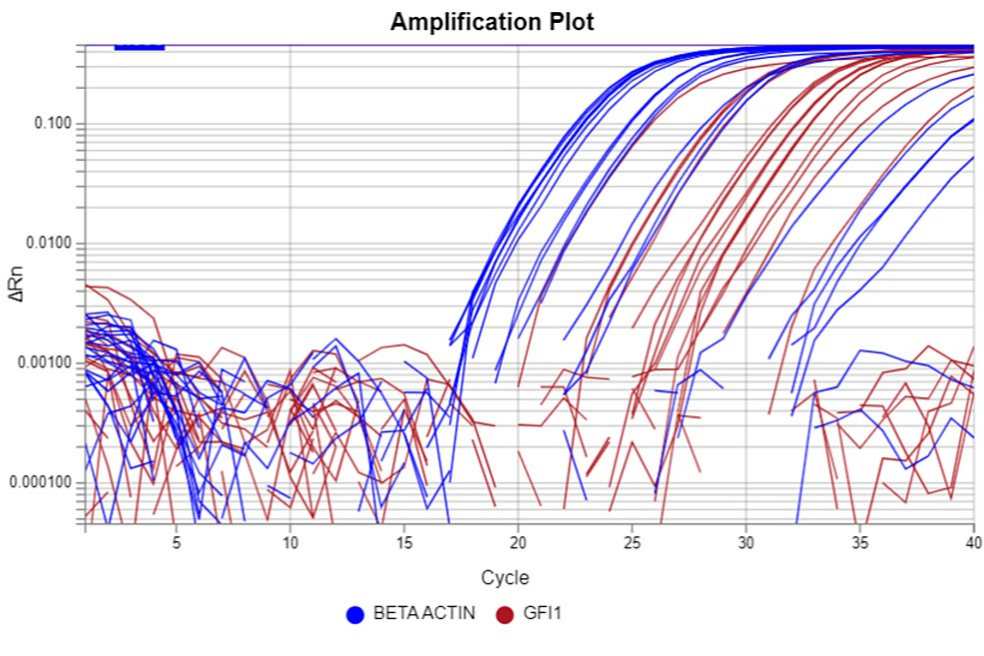
Amplification curve of the GFI-1 gene in a cord blood sample of a newborn during RT-PCR RT-PCR: real-time reverse transcriptase-polymerase chain reaction

In a genetic study, the GFI-1 gene was studied. The real-time reverse transcriptase-polymerase chain reaction (RT-PCR) was done, and the value is expressed as Delta CT. The amplification curve of the GFI-1 gene in a cord blood sample of a newborn during RT-PCR is shown in Figure 1.

**Figure 2:**
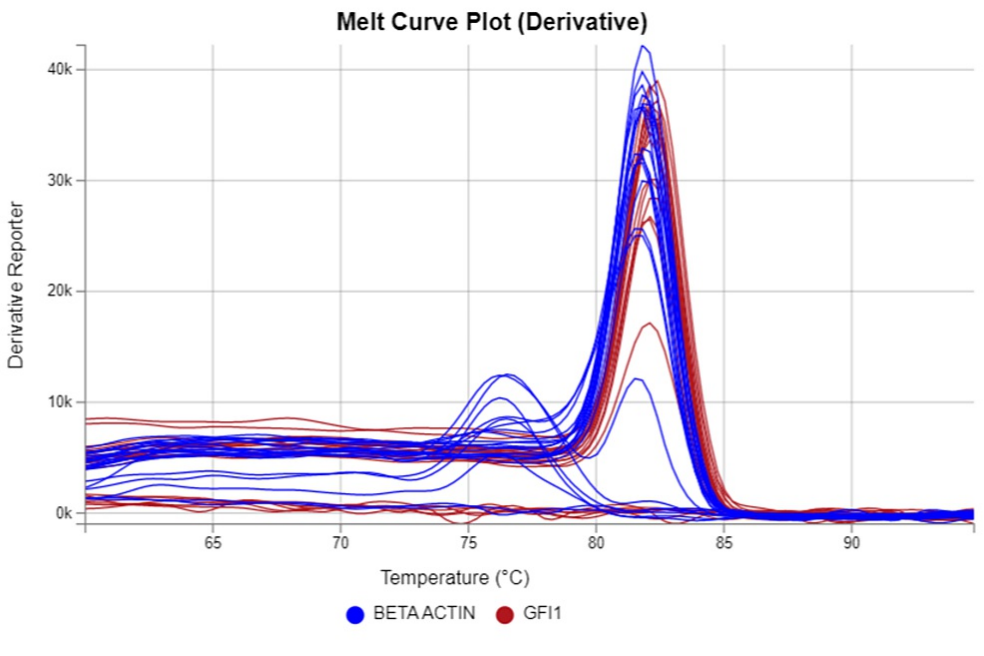
The melting curve of the GFI-1 gene in the cord blood samples of normal birth weight and low birth weight newborns during RT-PCR RT-PCR: real-time reverse transcriptase-polymerase chain reaction

The melting curve of the GFI-1 gene in cord blood samples from NBW and LBW newborns during RT-PCR is shown in Figure 2.

**Figure 3:**
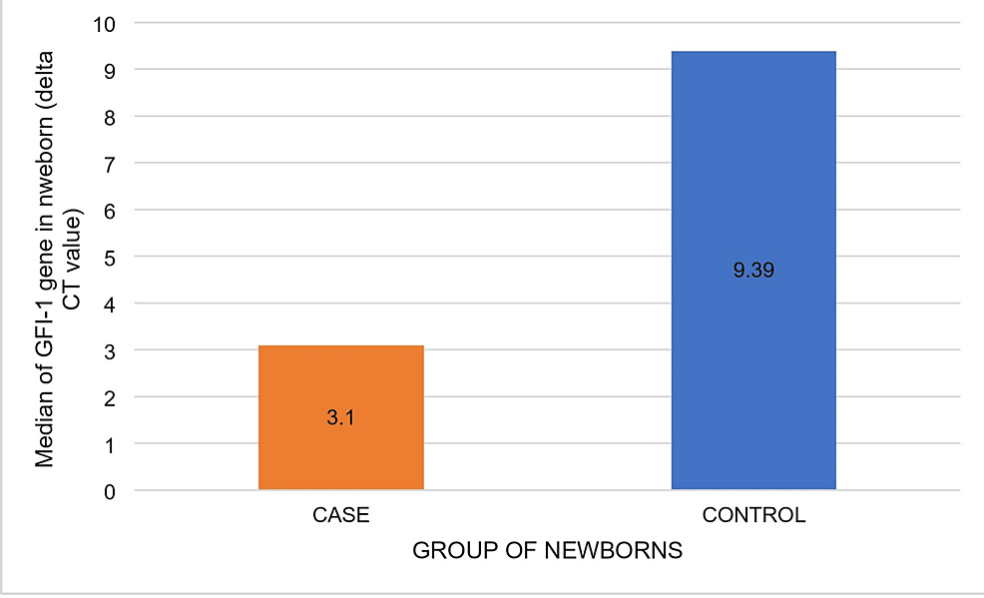
Expression of the GFI-1 gene (represented as the Delta CT value of quantitative RT-PCR) in the normal birth weight newborn group and the low birth weight newborn group. RT-PCR: real-time reverse transcriptase-polymerase chain reaction

**Figure 4:**
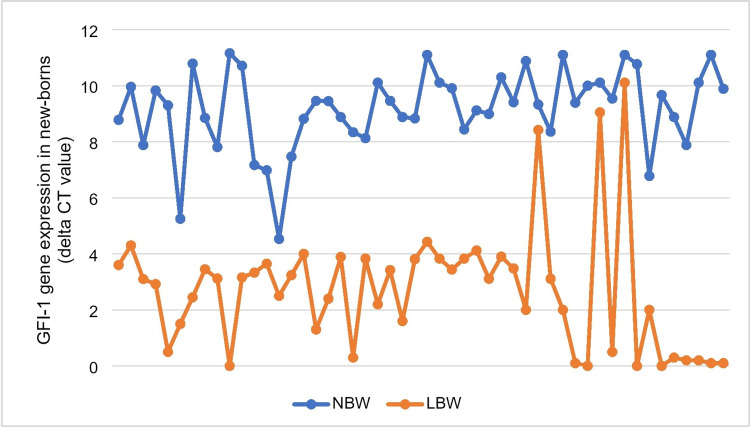
Comparison of the level of GFI -1 gene expression in low birth weight newborns and normal birth weight newborns

The median of GFI-1 expression (delta CT value of RT-PCR) in LBW newborns was 3.1, whereas it was 9.39 in NBW newborns. The difference was significant (p<0.001) (Figures 3-4 and Table 2).

**Table 2:** Level of the GFI-1 gene expression in low birth weight newborns and in normal birth weight newborns qPCR: quantitative polymerase chain reaction; Q1^st^: first quartile; Q3^rd^: third quartile

Groups	Delta CT value of GFI-1 gene in qPCR (mean ± SD)	Median with quartiles (Q1^st, ^Q3^rd^)	p-value
Low birth weight newborn (n=50)	2.7714 ± 2.20	3.10, (0.5, 3.81)	p<0.001
Normal birth weight newborn (n=50)	9.1820 ± 1.43	9.39, (8.44, 10.11)	

**Figure 5:**
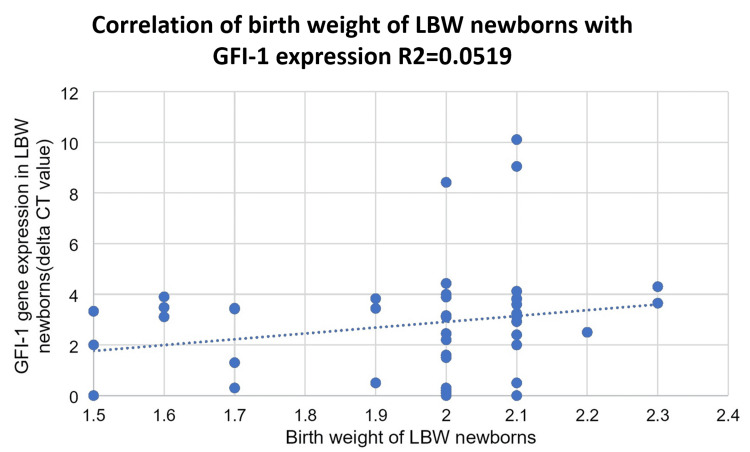
Correlation of the birth weight of low birth weight (LBW) newborns with the GFI-1 gene expression in term at birth

The level of the GFI-1 gene in LBW newborns was compared to the birth weight of newborns. The coefficient of correlation was found to be r = 0.223, which tells us that they are weakly, and positively correlated to each other (Figure 5).

**Figure 6:**
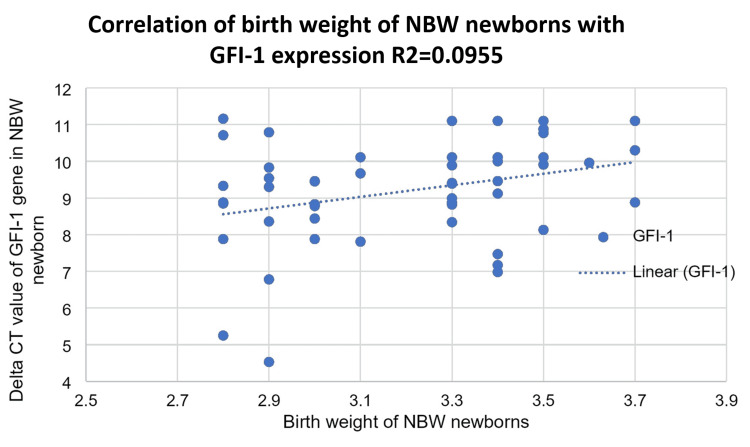
Correlation of the birth weight of normal birth weight (NBW) newborns with the GFI-1 expression in term at birth

The birth weight of NBW newborns was compared to the level of expression of the GFI-1 gene in NBW newborns at birth. The coefficient of correlation was found to be (r = 0.32). The birth weight of NBW newborns is positively correlated with GFI-1 gene expression (Figure 6).

## Discussion

The birth weight of an infant is the first weight recorded after birth, ideally measured within the first few hours after birth. The WHO defines LBW as a birth weight of less than 2500 grams (up to and including 2499 grams). The term "low birth weight" is defined as an absolute weight of <2500 grams after 37 completed gestational weeks. Small for gestational age (SGA) refers to newborns whose birth weight is less than the 10th percentile for gestational age. A VLBW is defined as a birth weight of <1500 grams and ELBW is defined as a birth weight of <1000 grams [23].

The incidence of LBW in India varies between 25% and 30%, of which 60% to 65% are due to IUGR [24]. The prevalence of LBW was highest in the central region of India (20.73%). Male newborns have a lower frequency of LBW than females. According to the London School of Hygiene and Tropical Medicine (LSHTM), The United Nations Children's Fund (UNICEF), and the WHO, every seventh baby born worldwide had LBW in 2015 [25]. In 2015, 20.5 million babies (14.6%) were found to have been born with LBW (less than 2.5 kg). While the prevalence in 2015 was lower than the 17.5% (22.9 million babies with LBW) in 2000, over 90% of the low-weight babies in 2015 were born in low- and middle-income countries [26].

Low birth weight remains a major cause of morbidity and mortality in low- and middle-income countries. It also has long-term effects on health outcomes in adult life. Low birth weight contributes to 60%-80% of all neonatal deaths [27].

This is a case-control study carried out on 100 patients (50 cases and 50 controls) with singleton pregnancies after 37 completed weeks of gestational age. Newborns with birth weights less than 2500 grams were taken as cases, and those with birth weights of 2500 grams and above were taken as controls.

In this study, we compared the expression of the GF-1 gene in hematopoietic stem cell differentiation in LBW and NBW babies after 37 completed weeks of gestational age. Our study shows a weak positive correlation (r = 0.223) between the GFI-1 gene and birth weight in LBW newborns as compared to NBW newborns, in whom there is more expression of GFI-1 (R = 0.332; P = 0.001), which is significant.

In the present study, maternal age was not found to be significant. In the present study, the occurrence of LBW was highest in the age group above 25 years (60%), similar to the study by Restrepo-Méndez MC et al. [28]. In another study, incidence was highest in mothers <17 years of age (3.2%) and gradually declined with advancing maternal age to reach 1.3% in women aged 25 to 34 years. It increased to 1.7% for those >35 years old. These findings may be due to socioeconomic inequalities rather than a younger maternal age [27, 28].

In the present analysis, the incidence of LBW was higher in multigravida women (72%; P = 0.001), similar to the meta-analysis by Bhattarai B. et al. [29], where the risk of LBW was higher in grand multiparous women compared to multiparous women. Primiparous and nulliparous women have less risk of having an LBW baby compared to multiparous women.

In our present analysis, the occurrence of LBW was 64% in lower socioeconomic status, compared to 28% in middle socioeconomic status, which is comparable and significant (P = 0.001), similar to the study by Mahmoodi et al. [30], which shows that low socioeconomic status increased the chance of delivering infants with LBW by 5.4 times (P <0.0001, odds ratio (OR) = 5.4). Working conditions had the highest direct and negative effect on birth weight, which shows that with more unfavorable working conditions, low birth weight is more likely to occur.

Our study showed that 36% of patients with LBW had matriculation as their educational status and 30% were illiterate, while in the control group, 38% had matriculation as their educational status and 26% were illiterate (P = 0.862), while the study done by Martinson et al. [31] shows that the rate of LBW was extremely high in the lowest educational category.

In our study, 30% of females with LBW newborns had a previous history of LBW, while only 10% of females with NBW newborns had a previous history of LBW. This is similar to the study done by Mvunta et al. [32], which showed the absolute recurrence rate of LBW was 28.1% with a relative risk of 5.08 fold.

In our study, the majority of cases (60%) had a BMI between 18.5 kg/m2 and 24.99 kg/m2, whereas the majority of controls (56%) had a BMI of 25 kg/m2 or above (P = 0.000). Lei Liu et al. [33] found that maternal underweight increased the risk of LBW (OR = 1.61, 95% CI 1.33-1.93) and was small for gestational age (OR = 1.75, 95% CI 1.51-2.02).

In our analysis, the majority of cases (56%) had a weight gain of up to 6 kg, while the majority of controls (98%) had a weight gain of 9 kg or more (P = 0.000). Sekine et al. [34] found that weight gain during the first trimester had a small effect on the risk of LBW and macrosomia. In the second trimester, insufficient weight gain was associated with the risk of LBW, and from the second trimester to delivery, a weight gain of less than 2 kg was associated with the risk of LBW.

In our analysis, concomitant systemic infections (urinary tract infection (UTI) and tuberculosis (TB)) were present in 14% of cases, while 6% of cases had infections, but that was not significant. Egbe et al. [35] found adverse fetal outcomes, including the presence of UTI, LBW, neonatal infection, and NICU admission. Mathad et al. [36] found that TB in the mother, as shown by the International Classification of Diseases, Clinical Modification (ICD-9) codes for the delivery discharge diagnosis, was strongly linked to a low birth weight.

In our present study, smoking was present in only 2% of cases, whereas in the control group, none had a history of smoking (P = 0.315). In our analysis, 4% of the cases had excess caffeine intake, whereas in the control group, 2% had excess caffeine intake, but that was not significant. Kataoka et al. [37] found the prevalence of smoking during pregnancy to be 13.4% in the study population. In full-term infants, birth weight decreased as the category of cigarettes per day increased.

In our study, vaginal bleeding in the first trimester was present in 8% of cases and 6% of the control group. Sun et al. [38] found that bleeding in the first trimester was related to an increased risk of LBW (risk ratio (RR) 2.52, 95% CI 1.34-4.75) and a 1.97-fold risk of small for gestational age (RR 1.97, 95% CI 1.19-3.25).

The major health problems associated with LBW include feeding difficulties, hypoglycemia, hypothermia, pulmonary immaturity, susceptibility to infection, and fluid and electrolyte imbalances. These problems require a higher rate of NICU admission in LBW babies than in NBW babies. In our study, 32% of babies with LBW required NICU admission, and 18% of NBW babies required NICU admission. Ballot et al. [39] reported that overall survival was 70.5%. Survival of infants below 1001 grams at birth was 34.9%, compared to 85.8% for those between 1001 and 1500 grams at birth. The main determinant of survival was birth weight, with an adjusted survival OR of 23.44 (95% CI: 11.22-49.00) for babies weighing between 1001 and 1500 grams compared to those weighing below 1001 g.

The APGAR score compared with NBW babies shows that LBW babies have a low APGAR score. In our study, 76% of cases had a one-minute APGAR score of eight, whereas in the control group, the APGAR score of eight at one minute was seen in 94% of cases. Six percent of LBW babies had an APGAR score of five, 2% had an APGAR score of six, and 16% had an APGAR score of seven at one minute (P=0.021). Lan et al. found that, based on the one minute of life test, the relative risks of LBW among infants with APGAR scores of 0 to three and four to six were 115.0 and 5.9 times higher, respectively, than those of normal infants. In the VLBW category, the relative risks of the above score were 252.5 and 51.1, in that order [40]. In the five-minute test of life, the relative risks of the above scores were 16.2 and 12.1 in the LBW category, respectively. In our study, despite the poor scores, 98% of LBW babies survived, while only 2% died.

The GFI-1 gene is the DNA-binding zinc finger transcription factor. It is the major regulator of both early hematopoiesis and hematopoietic stem cells. Its function is to act as a transcriptional repressor by recruiting histone-modifying enzymes to the promoters and enhancers of target genes. Both GFI-1 and GFI-1B have an important role in the endothelial cell-to-hematopoiesis transition, the process by which endothelial cells become blood cells during the third wave of blood development. Genes such as suppressor of cytokine signaling 3 (SOCS3), neutrophil elastase, colony-stimulating factor 1 (CSF1), and the CSF1 receptor, as well as the microRNAs miR-21 and miR-196b, are repressed by GFI-1 during myeloid cell development. Significant defects in neutrophil development in GFI-1-null mice and people with GIFI-1 mutations can be attributed to the downregulation of these genes. The GFI-1 gene has a C-terminal zinc-finger domain that can bind consensus DNA target sequences and an N-terminal SNAG domain that can suppress transcription in part by recruiting corepressors. GFI-1 is a transcriptional repressor that also binds to and inhibits STAT3 signaling via the protein inhibitor of the active signal transducer and activator of transcription (STAT). Hence, GFI-1 can counteract the inhibiting effects of active STAT3, allowing for more STAT3 signaling [41]. We predicted that GFI-1 would function as a regulator of cytokine and growth factor responses because STATs are activated by many different cytokines and growth factors.

In our study, we found that the expression of the GFI-1 gene is weakly positively correlated with the birth weight of LBW newborns (r = 0.223) and positively correlated (0.332) with the birth weight of NBW newborns. The difference in the expression of the GFI-1 gene between the two groups is significant (P <0.001). Miranda et al. concluded that considering the relation of IGF-I to growth and the decrease in difference in length, higher GFI-1 levels in VLBW infants in early childhood probably have an important role in catch-up growth in length [42].

Limitations

The major limitation of our study is the very low number of infants evaluated. Serial GFI-1 measurements from the early postnatal period to late childhood in both LBW and NBW newborns are needed to corroborate the results in larger samples of LBW children. The number of statistical analyses that can be conducted is also restricted. This means that it's important to be cautious when interpreting statistically significant results from a single experiment.

## Conclusions

The level of the GFI-1 gene and newborn birth weight were compared in LBW infants. They have a weakly positive correlation with one another. The level of GFI-1 gene expression at birth was compared to the birth weight of NBW newborns. The GFI-1 gene expression and birth weight are positively associated with NBW neonates.
